# Association of Mild Valvular Lesions With Long-term Cardiovascular Outcomes Among Black Adults

**DOI:** 10.1001/jamanetworkopen.2022.11946

**Published:** 2022-05-12

**Authors:** Kunihiro Matsushita, Yumin Gao, Jonathan Rubin, Ajay J. Kirtane, Susheel Kodali, Elizabeth Selvin, Alvaro Alonso, Martin B. Leon, Scott D. Solomon, Josef Coresh, Ervin R. Fox, Amil M. Shah

**Affiliations:** 1 Department of Epidemiology, Johns Hopkins Bloomberg School of Public Health, Baltimore, Maryland; 2 Division of Cardiology, Columbia University Irving Medical Center/New York-Presbyterian Hospital and the Cardiovascular Research Foundation, New York; 3 Department of Epidemiology, Rollins School of Public Health, Emory University, Atlanta, Georgia; 4 Division of Cardiovascular Medicine, Brigham and Women’s Hospital, Boston, Massachusetts; 5 Department of Medicine, University of Mississippi, Jackson

## Abstract

**Importance:**

Little is known about the long-term outcomes of mild valvular lesions.

**Objective:**

To examine the associations of 3 major types of valvular lesions (aortic stenosis, trace or mild aortic regurgitation, and trace or mild mitral regurgitation) with risk of cardiovascular mortality, coronary heart disease (CHD), stroke, heart failure, and atrial fibrillation.

**Design, Setting, and Participants:**

This cohort study analyzed data from the ongoing Atherosclerosis Risk in Communities study and focused on Black participants in the Jackson, Mississippi, site who underwent echocardiography at visit 3 from 1993 to 1995. Data analysis was conducted between April 2021 and February 2022.

**Exposures:**

Three valvular lesions were analyzed: aortic sclerosis, aortic regurgitation (trace or mild), and mitral regurgitation (trace or mild).

**Main Outcomes and Measures:**

The outcomes were cardiovascular mortality, coronary heart disease, heart failure, stroke, and atrial fibrillation. Multivariable Cox proportional hazards regression models were used to examine the independent associations between the 3 valvular lesions and these outcomes.

**Results:**

A total of 2106 Black participants were included, with a mean (SD) age of 59.1 (5.6) years and 1354 women (64.3%). The baseline prevalence was 7.7% for aortic sclerosis, 15.1% for aortic regurgitation (6.1% with trace, and 9.0% with mild), and 43.0% for mitral regurgitation (29.4% with trace, and 13.6% with mild). During a median (interquartile interval) follow-up of 22.5 (15.6-23.5) years, 890 participants developed at least 1 cardiovascular outcome. Each valvular lesion was significantly associated with at least 1 cardiovascular outcome: aortic sclerosis was associated with cardiovascular mortality (adjusted hazard ratio [HR], 1.54; 95% CI, 1.06-2.22), mild mitral regurgitation was associated with atrial fibrillation (HR, 1.47; 95% CI, 1.09-1.99), and trace or mild aortic regurgitation was associated with all outcomes (HRs ranging from 1.45 [95% CI, 1.17-1.81] to 1.75 [95% CI, 1.29-2.37]) except stroke. The total number of valvular lesions had graded associations with all cardiovascular outcomes except stroke: the HR of cardiovascular mortality was 1.77 (95% CI, 1.18-2.65) for those with 2 to 3 lesions and was 1.44 (95% CI, 1.05-1.96) for those with 1 lesion vs no lesions.

**Conclusions and Relevance:**

Results of this study indicate an association between valvular lesions, even at mild stage, and a long-term risk of cardiovascular events, suggesting the importance of recognizing and monitoring these valvular conditions.

## Key Points

**Question:**

Is there an association between mild valvular lesions, such as aortic sclerosis, aortic regurgitation, and mitral regurgitation, and cardiovascular outcomes?

**Findings:**

In this cohort study of 2106 Black participants in the Atherosclerosis Risk in Communities study, aortic sclerosis, trace or mild aortic regurgitation, and mild mitral regurgitation were each significantly associated with a long-term risk of cardiovascular events. A consistent dose-response association between the total number of valvular lesions and cardiovascular risk was also found.

**Meaning:**

Findings of this study suggest the importance of recognizing and monitoring individuals with valvular lesions.

## Teaser

This cohort study evaluates the risk of cardiovascular mortality, coronary heart disease, stroke, heart failure, and atrial fibrillation among Black individuals with aortic stenosis as well as trace or mild aortic regurgitation or mitral regurgitation.

## Introduction

Valvular heart disease (eg, aortic and mitral stenosis or regurgitation) is a major cause of heart failure and arrhythmias and increases the risk of cardiac mortality and morbidity.^1,2,3,4,5^ However, understanding of the potential implications of valvular heart disease for clinical outcomes is predominantly based on data from patients with advanced valvular heart disease that requires medical or surgical therapy,^6,7,8,9^ and the long-term outcome of mild valvular heart disease is yet to be determined.

To our knowledge, only 3 population-based studies of the long-term impact of valvular heart disease in the community have been conducted.^10,11,12^ However, there are several caveats regarding these studies. For example, 2 of the studies investigated only mortality as the outcome.^10,12^ Although mortality is an important outcome, to fully appreciate the prognostic value of valvular heart disease, it is necessary to study cardiovascular outcomes, including nonfatal cases. In addition, 1 of the 2 studies did not investigate mild valvular diseases.^10^ In this regard, Otto et al^11^ showed elevated risk of cardiovascular events (eg, myocardial infarction and heart failure) according to aortic sclerosis but did not study other valvular abnormalities.

Therefore, in this cohort study, we examined the associations of 3 major types of valvular lesions at mild stage (aortic sclerosis, trace or mild aortic regurgitation, and trace or mild mitral regurgitation) with risk of cardiovascular mortality, coronary heart disease (CHD), stroke, heart failure, and atrial fibrillation. We used nearly 25 years of follow-up data from the Atherosclerosis Risk in Communities (ARIC) study.

## Methods

### Study Design and Participants

The ARIC study was reviewed and approved by the institutional review board of each participating site. The relevant sites in the present study are Johns Hopkins Bloomberg School of Public Health, University of Mississippi, Wake Forest University, University of Minnesota, and University of North Carolina. Written informed consent was obtained from all participants. We followed the Strengthening the Reporting of Observational Studies in Epidemiology (STROBE) reporting guideline.^13^

The ARIC study is an ongoing community-based prospective cohort of 15 792 adults that began in 1987 with the enrollment of participants aged 45 to 64 years from 4 communities in the US: Forsyth County, North Carolina; Jackson, Mississippi; suburban Minneapolis, Minnesota; and Washington County, Maryland.^14^ Participants underwent a comprehensive medical examination from 1987 to 1989 (visit 1). Subsequent follow-up examinations were conducted from 1990 to 1992 (visit 2), 1993 to 1995 (visit 3), 1996 to 1998 (visit 4), 2011 to 2013 (visit 5), 2016 to 2017 (visit 6), and 2018 to 2019 (visit 7).

A total of 12 887 participants attended visit 3, but echocardiography was performed only at the Jackson site, wherein only Black participants were enrolled. Of the 2621 participants who attended visit 3 at the Jackson site, we excluded those with missing echocardiographic data (n = 99) or missing covariates of interest (n = 209) (eFigure 1 in the Supplement). We also excluded a small number of participants with aortic stenosis (n = 4), moderate or severe aortic regurgitation (n = 11), or moderate or severe mitral regurgitation (n = 16). The sample size of the primary analysis was 2106 participants.

Race data were self-identified by participants. The following categories were reported: American Indian or Alaskan Native, Asian, Black, and White.

### Echocardiographic Measurements

Two trained and certified technicians performed the echocardiographic examination using an echocardiogram machine (Acuson XP128/10c; Siemens Medical),^15^ which was equipped with three 128-element, dual-frequency, phased-array transducers. The primary operating frequencies of the V219 transducer (imaging at 2.5 MHz; Doppler at 2.0 MHz) or the V319 transducer (imaging at 3.5 MHz; Doppler at 2.5 MHz) were used for most echocardiographic studies. A series of 2-dimensional images of cardiac structure throughout the entire cardiac cycle and a full-screen acquisition of M-mode or spectral Doppler data were captured by a video-processing computer system (Freeland CineView; Freeland Systems). This system was also used to ensure image resolution and secure data transfer or storage. The quality control measures for echocardiography for visit 3 of the ARIC study have been previously described.^16,17^

Valvular lesions were assessed at the core reading center by 1 cardiologist who specialized in echocardiography. In this study, we focused on 3 relatively prevalent left-sided valvular lesions: aortic sclerosis, aortic regurgitation, and mitral regurgitation. Aortic sclerosis or stenosis was identified from echogenicity and thickening of the aortic leaflets as well as the presence or absence of stenosis and sclerosis distinguished by motion restriction of leaflets.^15,18^ Aortic regurgitations were assessed through visual assessment of the color Doppler signal. The ratio of the proximal jet height to the left ventricular outflow tract height was used to categorize aortic regurgitation into none, trace (≤5%), mild (>5% to ≤24%), moderate (≥25% to ≤46%), or severe (≥47%), as described previously.^16^ Mitral regurgitation was categorized in the same way, using the ratio of regurgitant jet area to atrial area, into none, trace (≤5%), mild (>5% to ≤20%), moderate (>20% to ≤40%), or severe (>40%). Aortic sclerosis, trace or mild aortic regurgitation, and trace or mild mitral regurgitation were the exposures of interest.

### Outcomes

The outcomes of interest were cardiovascular mortality, CHD, stroke, heart failure, and atrial fibrillation. Cardiovascular mortality consisted of deaths from CHD, stroke, or heart failure. Deaths were identified through contact with next of kin, search of hospital records and state death records, and linkage to the National Death Index. Coronary heart disease and stroke were defined as definite and probable cases as adjudicated by the ARIC study physician panel; this adjudication process was described previously.^19,20^ Heart failure was defined as either a hospitalization or death with a heart failure diagnosis with an *International Classification of Diseases, Ninth Revision* code 428 or an *International Statistical Classification of Diseases and Related Health Problems, Tenth Revision* code I50 in any position. Atrial fibrillation was defined as the first electrocardiogram showing atrial fibrillation or atrial flutter during study visits (as adjudicated by a trained cardiologist), as the first hospital discharge with atrial fibrillation with an *International Classification of Diseases, Ninth Revision* code 427.31 or 427.32 in any position, or as a cause of death.^21^ Participants without cardiovascular outcomes were followed up through December 31, 2017, date of death, or loss to follow-up, whichever occurred first.

We focused on incident cases of each cardiovascular disease (CVD) outcome by excluding participants with a history of relevant CVD subtype (eg, participants with prevalent CHD at baseline were excluded when incident CHD was analyzed). Thus, the analysis of cardiovascular mortality included all 2106 participants, but the analysis of other CVD outcomes included a smaller number of participants (as specified in the Results). The term *incident* was specific to each CVD subtype, and participants with multiple types of CVD outcomes during follow-up contributed to each relevant analysis as an outcome (eg, when a participant had CHD and then a stroke, both events were counted as incident CHD and incident stroke in relevant analyses, as appropriate).

### Covariates

The covariates of interest were assessed at visit 3, unless specified otherwise. Age, sex, and educational level (<high school, high school or vocational school diploma, or ≥college or graduate/professional school degree) were assessed at visit 1. Smoking status (current, former, or never) and use of medications (eg, antihypertensive and statin) were obtained from interviewer-administered questionnaires. Body mass index was calculated as weight in kilograms divided by height in meters squared. Diabetes was defined as a fasting glucose level of 126 mg/dL or higher, a nonfasting glucose level of 200 mg/dL or higher, a self-reported physician diagnosis of diabetes, or the use of antidiabetic medications. (To convert glucose levels to millimoles per liter, multiply by 0.0555.) Blood pressure was measured 3 times by a trained technician following a protocol, and the mean of the second and third readings was used in the analysis. Serum total cholesterol was measured using enzymatic assays, and high-density lipoprotein cholesterol was measured by a modified Lieberman Burchard method.^22^ Estimated glomerular filtration rate was calculated with the Chronic Kidney Disease Epidemiology Collaboration creatinine equation.^23^

History of CVD included a history of CHD, stroke, heart failure, or atrial fibrillation. History of CHD and stroke was defined either from self-report at visit 1 or from definite or probable events between visits 1 and 3 that were adjudicated by the ARIC study physician panel. History of heart failure was defined either from self-report at visit 1 or from hospitalization with heart failure between visits 1 and 3. History of atrial fibrillation was defined by review of a 12-lead electrocardiogram at visits 1 to 3 or as hospitalization with an atrial fibrillation diagnosis between visits 1 and 3.^24^

### Statistical Analysis

The baseline characteristics of participants were described by categories of aortic sclerosis, aortic regurgitation, and mitral regurgitation as mean (SD) for continuous variables and as count (percentage) for categorical variables. We estimated survival free of cardiovascular outcomes by categories of each valvular lesion using the Kaplan-Meier method, and then we compared those categories using the log-rank test.

We used multivariable Cox proportional hazards regression models to examine the independent associations of valvular lesions with cardiovascular outcomes, after adjustment for demographic characteristics and key clinical parameters of age, sex, educational level, body mass index, smoking status, diabetes, systolic blood pressure, antihypertensive medication use, total and high-density lipoprotein cholesterol levels, statin use, estimated glomerular filtration rate, and history of CHD, stroke, heart failure, and atrial fibrillation. In addition, we assessed the total number of valvular lesions in each participant (0, 1, or 2-3).

We conducted a sensitivity analysis by repeating the primary analysis but excluding participants with a history of CVD at baseline. We also explored whether the associations of valvular lesions with cardiovascular outcomes were independent of left ventricular systolic function, by adjusting for left ventricular fractional shortening (defined as the percentage difference in left ventricular diameter between systole and diastole). Given that aortic enlargement may precede aortic regurgitation and left ventricular dilation may precede mitral regurgitation, we also accounted for aortic root diameter for aortic regurgitation and left ventricular diameter for mitral regurgitation.

All statistical analyses were performed with Stata, version 15.1 (StataCorp LLC). A 2-sided *P* < .05 was considered statistically significant. Data analysis was conducted between April 2021 and February 2022.

## Results

A total of 2106 Black participants were included, with a mean (SD) age of 59.1 (5.6) years and with 1354 (64.3%) women and 752 (35.7%) men. The prevalence was 7.7% for aortic sclerosis (162 patients), 15.1% for aortic regurgitation (6.1% with trace [128 patients] and 9.0% with mild [190 patients]), and 43.0% for mitral regurgitation (29.4% with trace [620 patients] and 13.6% with mild [286 patients]). Compared with their counterparts, participants with aortic sclerosis, aortic regurgitation, or mitral regurgitation tended to be older and to have worse risk factor profiles, such as higher systolic blood pressure, lower kidney function, and a higher prevalence of previous CVD (Table 1). Compared with participants without aortic sclerosis, those with aortic sclerosis were also more likely to be current smokers and to have diabetes.

**Table 1.** Baseline Characteristics of Participants by Individual Valvular Lesions

Characteristic	No. (%)							
	Aortic sclerosis (N = 2106)		Aortic regurgitation (N = 2106)			Mitral regurgitation (N = 2106)		
	Without (n = 1944)	With (n = 162)	None (n = 1788)	Trace (n = 128)	Mild (n = 190)	None (n = 1200)	Trace (n = 620)	Mild (n = 286)
Age, mean (SD), y	58.7 (5.5)	63.4 (5.4)	58.8 (5.6)	60.0 (5.8)	61.0 (5.6)	58.7 (5.5)	59.4 (5.8)	60.1 (5.7)
Sex								
Female	1272 (65.4)	81 (50.0)	1169 (65.4)	68 (53.1)	116 (61.1)	749 (62.4)	408 (65.8)	196 (68.5)
Male	672 (34.6)	81 (50.0)	619 (34.6)	60 (46.9)	74 (38.9)	451 (37.6)	212 (34.2)	90 (31.5)
Educational level								
<High school	693 (35.6)	77 (47.5)	645 (36.1)	48 (37.5)	77 (40.5)	431 (35.9)	237 (38.2)	102 (35.7)
High school or vocational school diploma	548 (28.2)	43 (26.5)	509 (28.5)	27 (21.1)	55 (28.9)	348 (29.0)	168 (27.1)	75 (26.2)
≥College or graduate/professional school degree	703 (36.2)	42 (25.9)	634 (35.5)	53 (41.4)	58 (30.5)	421 (35.1)	215 (34.7)	109 (38.1)
BMI, mean (SD)	30.4 (6.2)	30.3 (6.2)	30.4 (6.2)	30.1 (5.5)	30.0 (6.6)	30.8 (6.3)	29.8 (6.1)	29.8 (5.8)
Smoking status								
Current	362 (18.6)	38 (23.5)	345 (19.3)	25 (19.5)	30 (15.8)	235 (19.6)	109 (17.6)	56 (19.6)
Former	612 (31.5)	66 (40.7)	573 (32.0)	42 (32.8)	63 (33.2)	387 (32.3)	206 (33.2)	85 (29.7)
Never	970 (49.9)	58 (35.8)	870 (48.7)	61 (47.7)	97 (51.1)	578 (48.2)	305 (49.2)	145 (50.7)
Diabetes	438 (22.5)	54 (33.3)	417 (23.3)	31 (24.2)	44 (23.2)	293 (24.4)	130 (21.0)	69 (24.1)
Antihypertensive medication use	967 (49.7)	114 (70.4)	895 (50.1)	68 (53.1)	118 (62.1)	618 (51.5)	302 (48.7)	161 (56.3)
Blood pressure, mean (SD), mm Hg								
Systolic	129.9 (19.6)	137.2 (22.0)	129.6 (19.6)	134.3 (19.3)	135.4 (21.9)	129.4 (19.8)	130.4 (19.6)	134.7 (20.5)
Diastolic	76.6 (10.4)	76.2 (11.6)	76.4 (10.3)	77.3 (11.3)	77.1 (11.4)	76.5 (10.3)	76.2 (10.6)	77.3 (11.0)
Cholesterol, mean (SD), mg/dL								
Total	206.3 (38.8)	213.2 (41.8)	206.8 (39.2)	210.1 (36.4)	205.1 (40.0)	207.8 (39.1)	205.3 (39.9)	206.4 (37.3)
HDL	56.2 (18.3)	51.1 (15.5)	55.7 (17.8)	56.2 (21.5)	56.5 (18.5)	55.8 (18.4)	56.0 (17.6)	55.3 (18.5)
Lipid-lowering medication use	83 (4.3)	14 (8.6)	78 (4.4)	11 (8.6)	8 (4.2)	53 (4.4)	33 (5.3)	11 (3.8)
eGFR, mean (SD), mL/min/1.73 m^2^	95.3 (19.3)	86.3 (22.1)	95.2 (19.5)	91.9 (19.6)	91.2 (21.2)	95.2 (19.4)	95.4 (18.5)	90.5 (22.7)
Prevalent CVD								
CHD	72 (3.7)	20 (12.3)	68 (3.8)	12 (9.4)	12 (6.3)	38 (3.2)	28 (4.5)	26 (9.1)
Stroke	45 (2.3)	8 (4.9)	44 (2.5)	5 (3.9)	4 (2.1)	31 (2.6)	14 (2.3)	8 (2.8)
Heart failure	108 (5.6)	20 (12.3)	100 (5.6)	3 (2.3)	25 (13.2)	61 (5.1)	39 (6.3)	28 (9.8)
Atrial fibrillation	8 (0.4)	0	7 (0.4)	1 (0.8)	0	5 (0.4)	3 (0.5)	0
LV, mean (SD)								
Fractional shortening, %	34.6 (8.8)	34.1 (10.5)	34.8 (8.9)	31.9 (8.9)	34.1 (9.5)	35.3 (8.6)	34.2 (8.8)	32.0 (10.0)
Septum thickness, cm	1.14 (0.22)	1.31 (0.30)	1.14 (0.22)	1.20 (0.25)	1.23 (0.31)	1.16 (0.24)	1.14 (0.22)	1.16 (0.24)
Diameter, cm	4.64 (0.58)	4.74 (0.63)	4.62 (0.58)	4.78 (0.54)	4.84 (0.67)	4.59 (0.57)	4.67 (0.56)	4.84 (0.69)
Posterior wall thickness, cm	1.14 (0.20)	1.24 (0.24)	1.14 (0.20)	1.19 (0.24)	1.21 (0.23)	1.16 (0.21)	1.14 (0.20)	1.17 (0.22)
Left atrium diameter, mean (SD), cm	3.85 (0.53)	3.99 (0.63)	3.85 (0.52)	3.83 (0.57)	3.93 (0.62)	3.85 (0.53)	3.83 (0.53)	3.98 (0.56)
Aortic sclerosis	0	162 (100)	89 (5.0)	29 (22.7)	44 (23.2)	68 (5.7)	57 (9.2)	37 (12.9)
Trace or mild aortic regurgitation	245 (12.6)	73 (45.1)	0	128 (100)	190 (100)	141 (11.8)	95 (15.3)	82 (28.7)
Trace or mild mitral regurgitation	812 (41.8)	94 (58.0)	729 (40.8)	72 (56.3)	105 (55.3)	0	620 (100)	286 (100)

Abbreviations: BMI, body mass index (calculated as weight in kilograms divided by height in meters squared); CHD, coronary heart disease; CVD, cardiovascular disease; eGFR, estimated glomerular filtration rate; HDL, high-density lipoprotein; LV, left ventricular.

SI conversion factor: To convert total and HDL cholesterol to mmol/L, multiply by 0.0259.

### Individual Valvular Lesions and Risk of Cardiovascular Outcomes

During a median (interquartile interval) follow-up of 22.5 (15.6-23.5) years, 890 of 2106 participants (42.3%) developed at least 1 of the cardiovascular outcomes. The most frequent cardiovascular outcome was heart failure (575 participants [27.3%]), followed by atrial fibrillation (306 [14.5%]), stroke (276 [13.1%]), CHD (237 [11.3%]), and cardiovascular mortality (222 [10.5%]).

**Figure 1.**
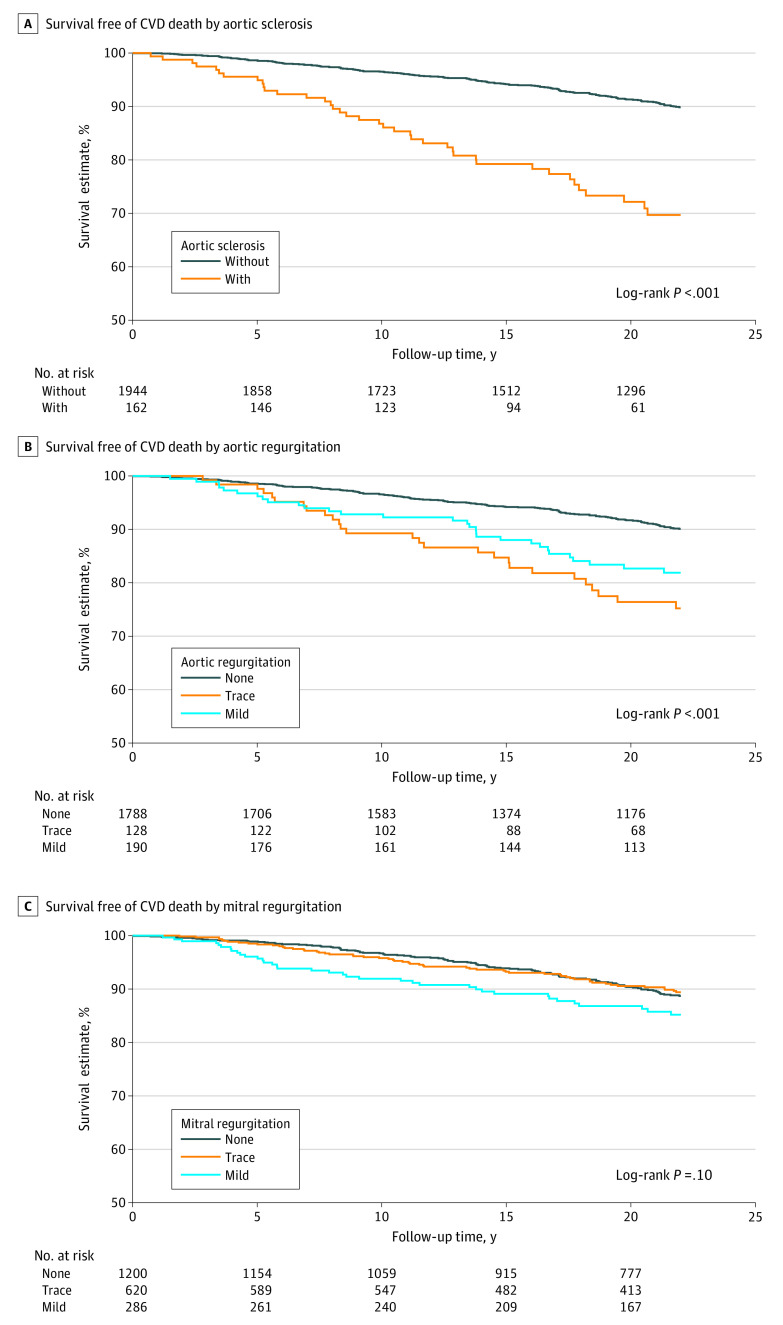
Kaplan-Meier Survival Estimates Free of Cardiovascular Disease (CVD) Death by Individual Valvular Lesions Cardiovascular mortality was defined as death from coronary heart disease, stroke, or heart failure.

When we compared survival free of cardiovascular mortality by valvular lesion type, we found that participants with aortic sclerosis, trace or mild aortic regurgitation, and mild mitral regurgitation had worse survival than their counterparts (Figure 1). For example, 20-year survival free of cardiovascular mortality was 72.1% for participants with aortic sclerosis compared with 91.3% for those without aortic sclerosis. Largely similar results were seen across other cardiovascular outcomes, but the results generally appeared most evident for heart failure and cardiovascular mortality across the 3 valvular lesion types (eFigures 2-4 in the Supplement).

According to the results in Figure 1, we combined trace or mild aortic regurgitation and none or trace mitral regurgitation in subsequent analyses. Even after the adjustment for potential confounders, each valvular lesion was independently associated with at least 1 outcome (Table 2). Aortic sclerosis demonstrated independent associations with cardiovascular mortality (adjusted hazard ratio [HR], 1.54; 95% CI, 1.06-2.22), and mitral regurgitation showed an association with atrial fibrillation (adjusted HR, 1.47; 95% CI, 1.09-1.99). Aortic regurgitation showed independent associations with all individual outcomes, with adjusted HRs ranging from 1.45 (95% CI, 1.17-1.81) to 1.75 (95% CI, 1.29-2.37), except for stroke. The results remained largely consistent after participants with a history of CVD at baseline were excluded (eTable 1 in the Supplement). Further adjustment for additional relevant echocardiographic parameters did not materially change the results (eTable 2 in the Supplement).

**Table 2.** Adjusted Hazard Ratios of Cardiovascular Outcomes by Individual Valvular Lesions^a^

Cardiovascular outcome	Adjusted hazard ratio (95% CI)					
	Aortic sclerosis		Aortic regurgitation		Mitral regurgitation	
	Without	With	None	Trace or mild	None or trace	Mild
Cardiovascular mortality	1 [Reference]	1.54 (1.06-2.22)^b^	1 [Reference]	1.75 (1.29-2.37)^c^	1 [Reference]	1.08 (0.75-1.55)
CHD	1 [Reference]	1.49 (0.95-2.34)	1 [Reference]	1.55 (1.08-2.21)^b^	1 [Reference]	1.09 (0.73-1.64)
Stroke	1 [Reference]	1.31 (0.88-1.96)	1 [Reference]	0.89 (0.63-1.26)	1 [Reference]	1.10 (0.78-1.56)
Heart failure	1 [Reference]	1.08 (0.80-1.45)	1 [Reference]	1.45 (1.17-1.81)^c^	1 [Reference]	1.15 (0.90-1.48)
Atrial fibrillation	1 [Reference]	0.85 (0.57-1.29)	1 [Reference]	1.47 (1.11-1.94)^d^	1 [Reference]	1.47 (1.09-1.99)^b^

Abbreviation: CHD, coronary heart disease.

^a^ Models were adjusted for age, sex, educational level, body mass index, smoking status, diabetes, antihypertensive medication use, systolic blood pressure, total and high-density lipoprotein cholesterol, statin use, estimated glomerular filtration rate, and history of CHD, stroke, heart failure, or atrial fibrillation. For example, in the analysis of incident CHD, those with a history of CHD were excluded, and the model was adjusted for history of stroke, heart failure, and atrial fibrillation. The numbers of participants who were excluded from the relevant analysis because of prevalent cases of relevant cardiovascular disease subtypes at baseline were 92 for CHD, 53 for stroke, 128 for heart failure, and 8 for atrial fibrillation.

^b^ *P* < .05.

^c^ *P* < .001.

^d^ *P* < .01.

### Total Number of Valvular Lesions and Cardiovascular Outcomes

A total of 146 participants (6.9%) had 2 to 3 lesions (23 [1.1%] had 3 lesions), and 451 (21.4%) had 1 lesion. The total number of valvular lesions demonstrated dose-response associations with all of the cardiovascular outcomes tested, although stroke did not reach statistical significance (Table 3). For example, those with 2 to 3 lesions had an adjusted HR of cardiovascular mortality of 1.77 (95% CI, 1.18-2.65) and those with 1 lesion had an HR of 1.44 (95% CI, 1.05-1.96). The HR for 2 to 3 lesions vs no lesions was 1.88 (95% CI, 1.20-2.94) for CHD and 1.54 (95% CI, 1.05-2.27) for atrial fibrillation. Again, the association patterns were similar after the exclusion of participants with a history of CVD at baseline (eTable 3 in the Supplement).

**Table 3.** Adjusted Hazard Ratios of Cardiovascular Outcomes by Number of Valvular Lesions^a^

Cardiovascular outcome	No. of valvular lesions, adjusted hazard ratio (95% CI)		
	0	1	≥2
Total participants, No. (%)	1509 (71.7)	451 (21.4)	146 (6.9)
Cardiovascular mortality	1 [Reference]	1.44 (1.05-1.96)^b^	1.77 (1.18-2.65)^c^
CHD	1 [Reference]	1.02 (0.71-1.48)	1.88 (1.20-2.94)^c^
Stroke	1 [Reference]	1.03 (0.76-1.40)	1.17 (0.75-1.82)
Heart failure	1 [Reference]	1.26 (1.02-1.56)^b^	1.48 (1.10-1.99)^c^
Atrial fibrillation	1 [Reference]	1.30 (0.99-1.71)	1.54 (1.05-2.27)^b^

Abbreviation: CHD, coronary heart disease.

^a^ Models were adjusted for age, sex, educational level, body mass index, smoking status, diabetes, antihypertensive medication use, systolic blood pressure, total and high-density lipoprotein cholesterol, statin use, estimated glomerular filtration rate, and history of CHD, stroke, heart failure, or atrial fibrillation. For example, in the analysis of incident CHD, those with a history of CHD were excluded, and the model was adjusted for history of stroke, heart failure, and atrial fibrillation. The numbers of participants who were excluded from the relevant analysis because of prevalent cases of relevant cardiovascular disease subtypes at baseline were 92 for CHD, 53 for stroke, 128 for heart failure, and 8 for atrial fibrillation.

^b^ *P* < .05.

^c^ *P* < .01.

**Figure 2.**
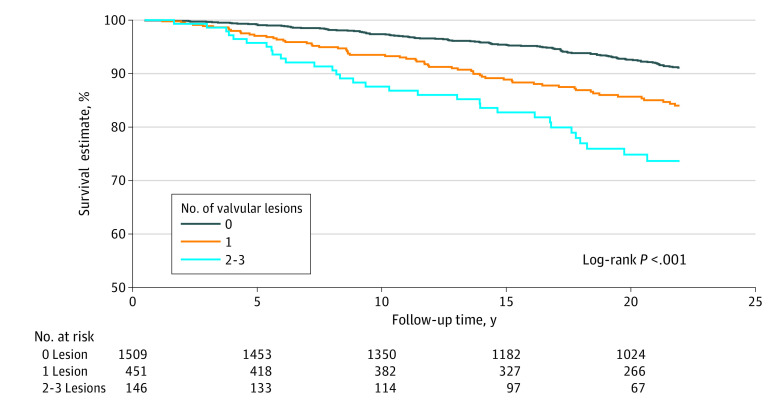
Kaplan-Meier Survival Estimates Free of Cardiovascular Disease Death by Number of Valvular Lesions Valvular lesions were defined as aortic sclerosis, trace or mild aortic regurgitation, and mild mitral regurgitation, based on their independent associations with cardiovascular disease death. Cardiovascular mortality was defined as death from coronary heart disease, stroke, or heart failure.

In terms of absolute risk, 20-year survival free of cardiovascular mortality was 74.9% in participants with 2 to 3 lesions, 85.7% in participants with 1 lesion, and 92.6% in participants with no lesions (Figure 2). The patterns were similar for other outcomes (eFigure 5 in the Supplement).

## Discussion

In this prospective cohort study of middle-aged Black adults, we observed that 3 types of mild valvular lesions (aortic sclerosis, aortic regurgitation, and mitral regurgitation) were associated with adverse cardiovascular outcomes over approximately 25 years in the ARIC study. Even after the adjustment for potential confounders, each valvular lesion remained associated with at least 1 of the cardiovascular outcomes: aortic sclerosis was associated with cardiovascular mortality, trace or mild aortic regurgitation was associated with all outcomes except stroke, and mild mitral regurgitation was associated with atrial fibrillation. When the total number of valvular lesions was assessed, there was a consistent dose-response association with cardiovascular risk. The results were largely consistent after excluding participants with prevalent CVD at baseline. Further adjustment for other relevant echocardiographic parameters (eg, fractional shortening) did not materially alter the results. Of note, approximately 30% of the study participants had at least 1 valvular lesion, which was a factor in a potentially poor outcome.

These findings are generally in agreement with results of 3 previous community-based studies that showed an association between valvular lesions and a poor long-term outcome.^10,11,12^ Nonetheless, the present study has a few unique aspects. First, to our knowledge, this study was the first to show the long-term implications of mild aortic regurgitation and mild mitral regurgitation individually. Second, we investigated lesion-specific associations with several major cardiovascular outcomes. Third, we rigorously adjusted for potential confounders, including adiposity, blood pressure, and kidney function. Fourth, we found a graded risk of cardiovascular outcomes according to the total number of affected valvular lesions.

Valvular heart disease (especially at moderate and severe stages) is a known cause of cardiac remodeling and hemodynamic changes.^25,26,27^ Whether and to what extent these pathophysiological mechanisms play a role in the long-term outcome of mild valvular lesions are uncertain and should be explored in future studies. Nonetheless, the results for individual outcomes in this study seemed to reflect the current knowledge about the pathophysiological mechanisms of specific valvular lesions. For example, mild mitral regurgitation was most robustly associated with incident atrial fibrillation. In addition, it is possible that these results reflect the shared risk factors between valvular lesions and cardiovascular outcomes, although we accounted for potential confounders.

Among the 3 valvular lesions tested, trace or mild aortic regurgitation had the most robust association with cardiovascular outcomes. This finding may reflect the different hemodynamic changes on the left ventricle associated with these valvular lesions. For example, sole aortic sclerosis would be associated with minimal hemodynamic impact. Mitral regurgitation can affect the left atrium at an early stage but may need to be at a moderate or severe stage to be a factor in left ventricular function and structure. In contrast, aortic regurgitation may be associated with increased left ventricular end-diastolic pressure and thus alter both the left ventricle and the left atrium. Nonetheless, future studies are needed to confirm this observation of aortic regurgitation being a precursor of various cardiovascular outcomes and to identify potential cardiac remodeling processes using longitudinal echocardiographic measures.

Historically, clinical attention to valvular heart disease has focused on its moderate to severe stages^28^; the present study, however, examined mild valvular lesions. Clinical guidelines typically address each valvular disease,^28,29,30^ but this study highlighted mixed valvular lesions, even when each lesion was mild. The results suggest monitoring individuals with mild valvular lesions, particularly when some lesions coexist, for further progression and for any modifiable traditional risk factors. The absolute risk difference between the presence or absence of valvular lesions was not small. The 20-year survival free of cardiovascular mortality was 74.9% with 2 to 3 valvular lesions vs 92.6% with no lesions.

Although this study relied on jet area to assess regurgitation (which was a standard approach at the time of the cohort baseline visit [1993-1995]), current clinical guidelines emphasize taking into account multiple parameters, such as valve hemodynamics, valve anatomy, and the role of valvular disease in cardiac structure and function, when evaluating valvular heart disease.^30,31^ Such an integrated approach suggests the potential value of using artificial intelligence or machine learning to leverage the full range of information obtained through echocardiograms for the beneficial and efficient care of persons with valvular lesions.

### Limitations

This study has several limitations. First, only middle-aged Black participants were included in the study, making the generalizability of these results to other racial and ethnic groups uncertain. For example, a lower burden of aortic stenosis in Black than in White individuals has been reported.^32^ Nonetheless, the data that were specifically about Black individuals are valuable given that this group experiences disparities in clinical care and research on valvular heart disease.^33^ Second, heart failure and atrial fibrillation were not adjudicated by the physician panel, yet high positive predictive values for both outcomes have been demonstrated in previous analyses of the ARIC study.^34,35^ Third, given the number of analyses we conducted, we cannot deny the possibility of chance findings. Nonetheless, we considered the strength and robustness of association, dose-response association, and biological plausibility, and not only the statistical significance of the data. Fourth, a relatively small sample size precluded our ability to conduct subgroup analysis. Fifth, as with all observational studies, we cannot exclude the possibility of residual confounding.

## Conclusions

This cohort study found that aortic sclerosis, trace or mild aortic regurgitation, and mild mitral regurgitation were each significantly associated with a long-term risk of cardiovascular events in Black individuals. The results of this study suggest the importance of recognizing and monitoring individuals with these valvular conditions.

## Notes

Supplement. **eTable 1.** Adjusted Hazard Ratios of Cardiovascular Outcomes by Individual Valvular Lesions When Excluding Those With a History of Cardiovascular Disease at Baseline **eTable 2.** Adjusted Hazard Ratios of Cardiovascular Outcomes by Individual Valvular Lesions After Adjusting for Echocardiographic Measures **eTable 3.** Adjusted Hazard Ratios of Cardiovascular Outcomes by the Number of Valvular Lesions When Excluding Those With a History of Cardiovascular Disease at Baseline **eFigure 1.** Study Participant Flow Diagram **eFigure 2.** Kaplan-Meier Survival Estimates of Cardiovascular Outcomes by Aortic Sclerosis **eFigure 3.** Kaplan-Meier Survival Estimates of Cardiovascular Outcomes by Aortic Regurgitation **eFigure 4.** Kaplan-Meier Survival Estimates of Cardiovascular Outcomes by Mitral Regurgitation **eFigure 5.** Kaplan-Meier Survival Estimates of Cardiovascular Outcomes by the Number of Valvular Lesions
