# Supplementary material for: Association of Mild Valvular Lesions With Long-term Cardiovascular Outcomes Among Black Adults
Source: JAMA Netw Open. 2022 May 12;5(5):e2211946. doi: 10.1001/jamanetworkopen.2022.11946 (PMC9099428; doi:10.1001/jamanetworkopen.2022.11946)
Supplement: Supplement. — eTable 1. Adjusted Hazard Ratios of Cardiovascular Outcomes by Individual Valvular Lesions When Excluding Those With a History of Cardiovascular Disease at Baseline eTable 2. Adjusted Hazard Ratios of Cardiovascular Outcomes by Individual Valvular Lesions After Adjusting for Echocardiographic Measures eTable 3. Adjusted Hazard Ratios of Cardiovascular Outcomes by the Number of Valvular Lesions When Excluding Those With a History of Cardiovascular Disease at Baseline eFigure 1. Study Participant Flow Diagram eFigure 2. Kaplan-Meier Survival Estimates of Cardiovascular Outcomes by Aortic Sclerosis eFigure 3. Kaplan-Meier Survival Estimates of Cardiovascular Outcomes by Aortic Regurgitation eFigure 4. Kaplan-Meier Survival Estimates of Cardiovascular Outcomes by Mitral Regurgitation eFigure 5. Kaplan-Meier Survival Estimates of Cardiovascular Outcomes by the Number of Valvular Lesions [file jamanetwopen-e2211946-s001.pdf]

Supplementary Online Content

Matsushita K, Gao Y, Rubin J, et al. Association of mild valvular lesions with long-term cardiovascular outcomes among Black adults. *JAMA Netw Open*. 2022;5(5):e2211946. doi:10.1001/jamanetworkopen.2022.11946

eTable 1. Adjusted Hazard Ratios of Cardiovascular Outcomes by Individual Valvular Lesions When Excluding Those With a History of Cardiovascular Disease at Baseline

eTable 2. Adjusted Hazard Ratios of Cardiovascular Outcomes by Individual Valvular Lesions After Adjusting for Echocardiographic Measures

eTable 3. Adjusted Hazard Ratios of Cardiovascular Outcomes by the Number of Valvular Lesions When Excluding Those With a History of Cardiovascular Disease at Baseline

eFigure 1. Study Participant Flow Diagram

eFigure 2. Kaplan-Meier Survival Estimates of Cardiovascular Outcomes by Aortic Sclerosis

eFigure 3. Kaplan-Meier Survival Estimates of Cardiovascular Outcomes by Aortic Regurgitation

eFigure 4. Kaplan-Meier Survival Estimates of Cardiovascular Outcomes by Mitral Regurgitation

eFigure 5. Kaplan-Meier Survival Estimates of Cardiovascular Outcomes by the Number of Valvular Lesions

This supplementary material has been provided by the authors to give readers additional information about their work.

© 2022 Matsushita K et al. *JAMA Network Open.*

eTable 1. Adjusted Hazard Ratios of Cardiovascular Outcomes by Individual Valvular Lesions When Excluding Those With a History of Cardiovascular Disease at Baseline (N=1862)

**Cardiovascular Outcomes**	**Valvular Lesions, Hazard Ratio (95% CI)**					
	**Aortic Sclerosis**		**Aortic Regurgitation**		**Mitral Regurgitation**	
	**None**	**Present**	**None**	**Trace/Mild**	**None/Trace**	**Mild**
Cardiovascular mortality	1 [Reference]	1.79 (1.15-2.78)[a]	1 [Reference]	1.56 (1.08-2.26)[a]	1 [Reference]	1.00 (0.63-1.58)
Coronary heart disease	1 [Reference]	1.66 (1.04-2.65)[a]	1 [Reference]	1.72 (1.19-2.48)[b]	1 [Reference]	1.11 (0.73-1.70)
Stroke	1 [Reference]	1.36 (0.86-2.14)	1 [Reference]	0.93 (0.64-1.37)	1 [Reference]	1.17 (0.80-1.71)
Heart failure	1 [Reference]	1.08 (0.79-1.49)	1 [Reference]	1.30 (1.02-1.64)[a]	1 [Reference]	1.10 (0.84-1.43)
Atrial fibrillation	1 [Reference]	0.98 (0.61-1.56)	1 [Reference]	1.56 (1.15-2.13)[b]	1 [Reference]	1.59 (1.15-2.20)[b]

[a] $p < 0.05$
[b] $p < 0.01$

Models were adjusted for the following covariates: age, gender, education, body mass index, smoking status, diabetes, anti-hypertensive use, systolic blood pressure, total and high-density lipoprotein (HDL) cholesterols, statin use, and estimated glomerular filtration rate (eGFR).

© 2022 Matsushita K et al. *JAMA Network Open.*

eTable 2. Adjusted Hazard Ratios of Cardiovascular Outcomes by Individual Valvular Lesions After Adjusting for Echocardiographic Measures

Cardiovascular Outcomes	Valvular Lesions, Hazard Ratio (95% CI)					
	Aortic Sclerosis		Aortic Regurgitation		Mitral Regurgitation	
	None	Present	None	Trace/Mild	None/Trace	Mild
**Model A[d]**						
Cardiovascular mortality	1 [Reference]	1.61 (1.08-2.39)[a]	1 [Reference]	1.80 (1.29-2.50)[c]	1 [Reference]	0.91 (0.60-1.37)
Coronary heart disease	1 [Reference]	1.56 (0.96-2.53)	1 [Reference]	1.47 (0.99-2.16)	1 [Reference]	1.01 (0.65-1.57)
Stroke	1 [Reference]	1.29 (0.85-1.96)	1 [Reference]	0.83 (0.57-1.22)	1 [Reference]	1.06 (0.73-1.54)
Heart failure	1 [Reference]	1.14 (0.84-1.55)	1 [Reference]	1.44 (1.14-1.82)[b]	1 [Reference]	1.04 (0.79-1.36)
Atrial fibrillation	1 [Reference]	0.91 (0.59-1.41)	1 [Reference]	1.43 (1.05-1.94)[a]	1 [Reference]	1.40 (1.02-1.94)[a]
**Model B[e]**						
Cardiovascular mortality	-	-	1 [Reference]	1.65 (1.18-2.31)[b]	-	-
Coronary heart disease	-	-	1 [Reference]	1.31 (0.88-1.95)	-	-
Stroke	-	-	1 [Reference]	0.91 (0.63-1.32)	-	-
Heart failure	-	-	1 [Reference]	1.36 (1.07-1.73)[a]	-	-
Atrial fibrillation	-	-	1 [Reference]	1.44 (1.06-1.96)[a]	-	-
**Model C[f]**						
Cardiovascular mortality	-	-	-	-	1 [Reference]	0.91 (0.60-1.37)
Coronary heart disease	-	-	-	-	1 [Reference]	1.02 (0.66-1.59)
Stroke	-	-	-	-	1 [Reference]	1.09 (0.75-1.59)
Heart failure	-	-	-	-	1 [Reference]	0.99 (0.76-1.31)
Atrial fibrillation	-	-	-	-	1 [Reference]	1.46 (1.06-2.03)[a]

© 2022 Matsushita K et al. *JAMA Network Open.*

[a] $p < 0.05$
[b] $p < 0.01$
[c] $p < 0.001$
[d] Model A: additionally adjusting for fractional shortening of left ventricle on top of the main model (N=1870)
[e] Model B: additionally adjusting for diameter of aortic root on top of the main model (N=1788)
[f] Model C: additionally adjusting for left ventricular diameter on top of the main model (N=1880)

Models were adjusted for the following covariates: age, gender, education, body mass index, smoking status, diabetes, anti-hypertensive use, systolic blood pressure, total and high-density lipoprotein (HDL) cholesterols, statin use, estimated glomerular filtration rate (eGFR), and history of coronary heart disease, stroke, heart failure, or atrial fibrillation.

© 2022 Matsushita K et al. *JAMA Network Open.*

eTable 3. Adjusted Hazard Ratios of Cardiovascular Outcomes by the Number of Valvular Lesions When Excluding Those With a History of Cardiovascular Disease at Baseline (N=1862)

**Cardiovascular Outcomes**	**Number of Valvular Lesions, Hazard Ratio (95% CI)**		
	**0**	**1**	**≥2**
Total participants, No.	1370	378	114
Cardiovascular mortality	1 [Reference]	1.51 (1.05-2.17)[a]	1.58 (0.95-2.65)
Coronary heart disease	1 [Reference]	1.05 (0.71-1.55)	2.16 (1.37-3.42)[b]
Stroke	1 [Reference]	1.00 (0.72-1.41)	1.32 (0.81-2.14)
Heart failure	1 [Reference]	1.23 (0.98-1.54)	1.33 (0.96-1.83)
Atrial fibrillation	1 [Reference]	1.36 (1.00-1.84)[a]	1.82 (1.20-2.77)[b]

[a] p <0.05
[b] p <0.01

Models were adjusted for the following covariates: age, gender, education, body mass index, smoking status, diabetes, anti-hypertensive use, systolic blood pressure, total and high-density lipoprotein (HDL) cholesterols, statin use, and estimated glomerular filtration rate (eGFR).

© 2022 Matsushita K et al. *JAMA Network Open.*

eFigure 1. Study Participant Flow Diagram

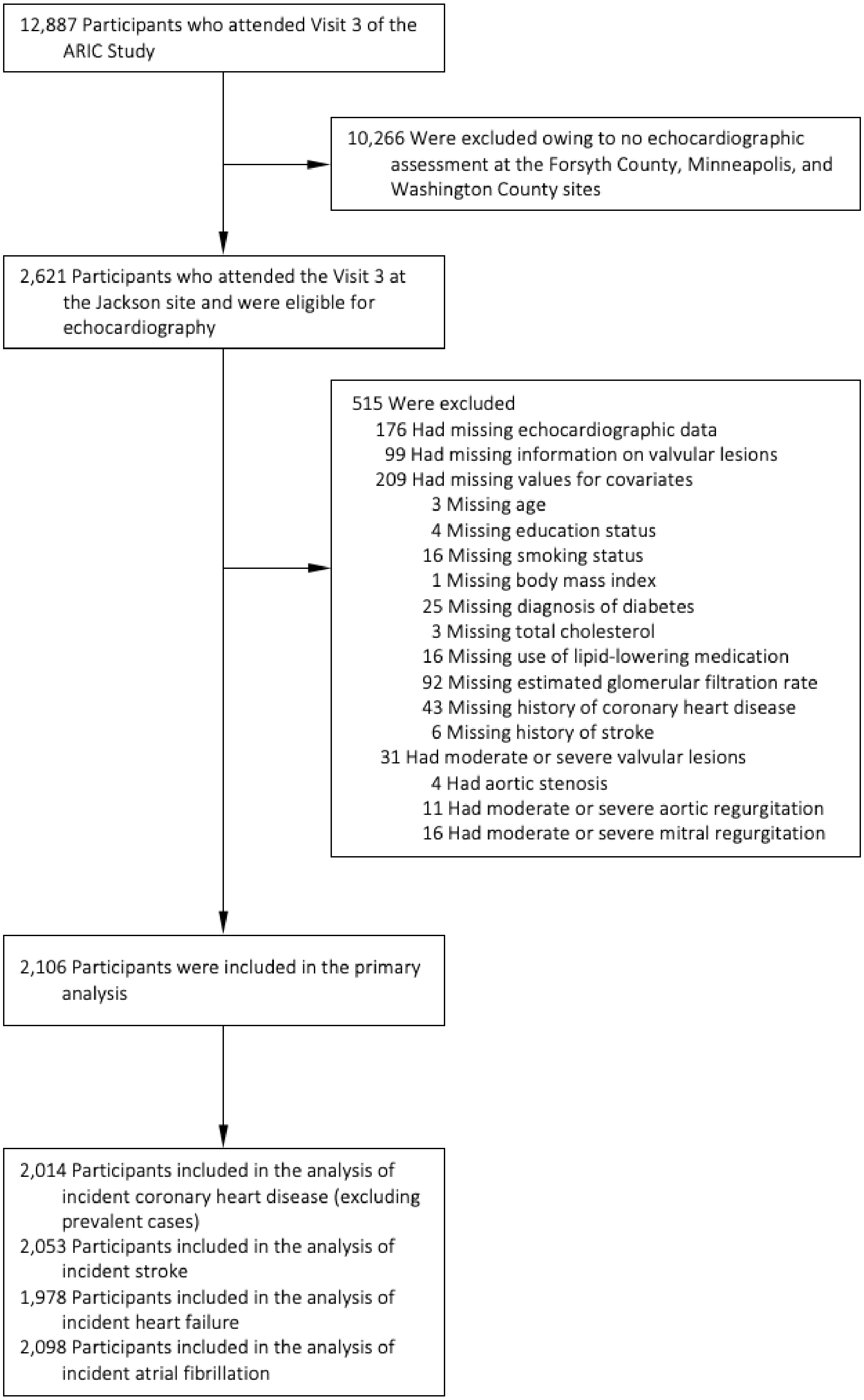

© 2022 Matsushita K et al. *JAMA Network Open.*

eFigure 2. Kaplan-Meier Survival Estimates of Cardiovascular Outcomes by Aortic Sclerosis

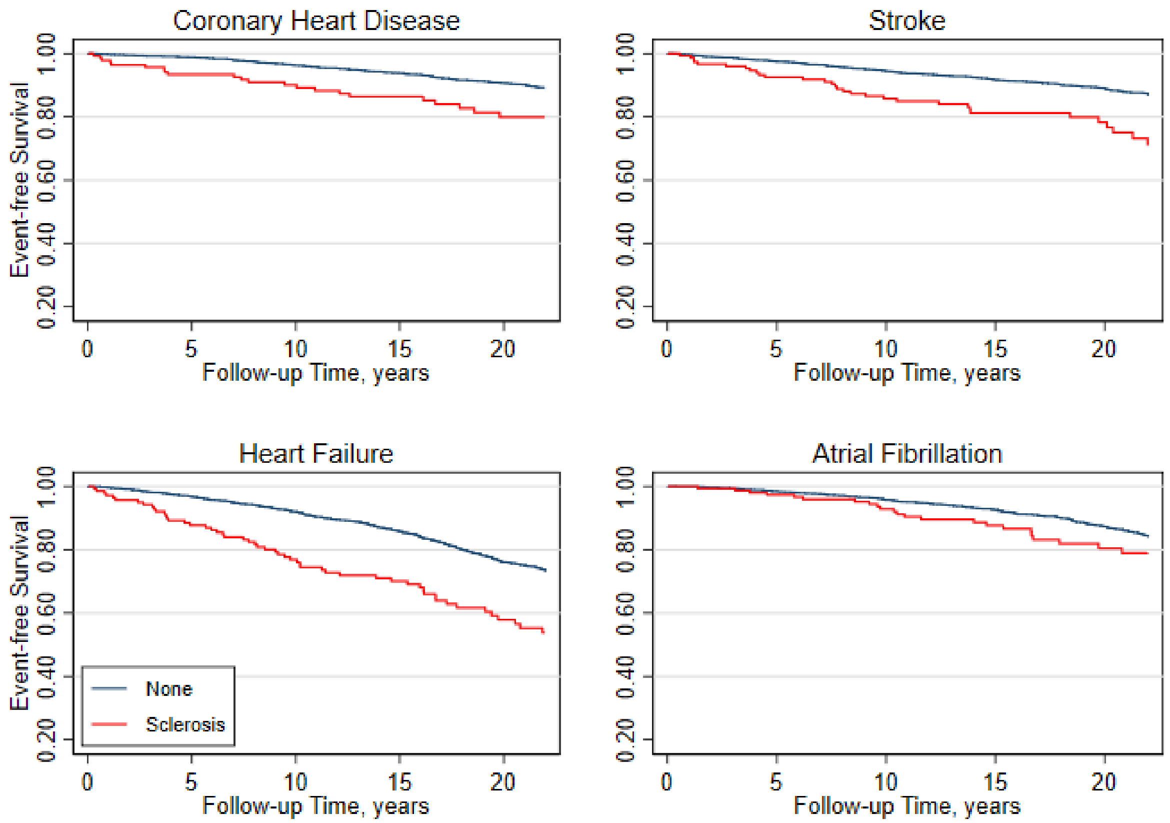

© 2022 Matsushita K et al. *JAMA Network Open.*

eFigure 3. Kaplan-Meier Survival Estimates of Cardiovascular Outcomes by Aortic Regurgitation

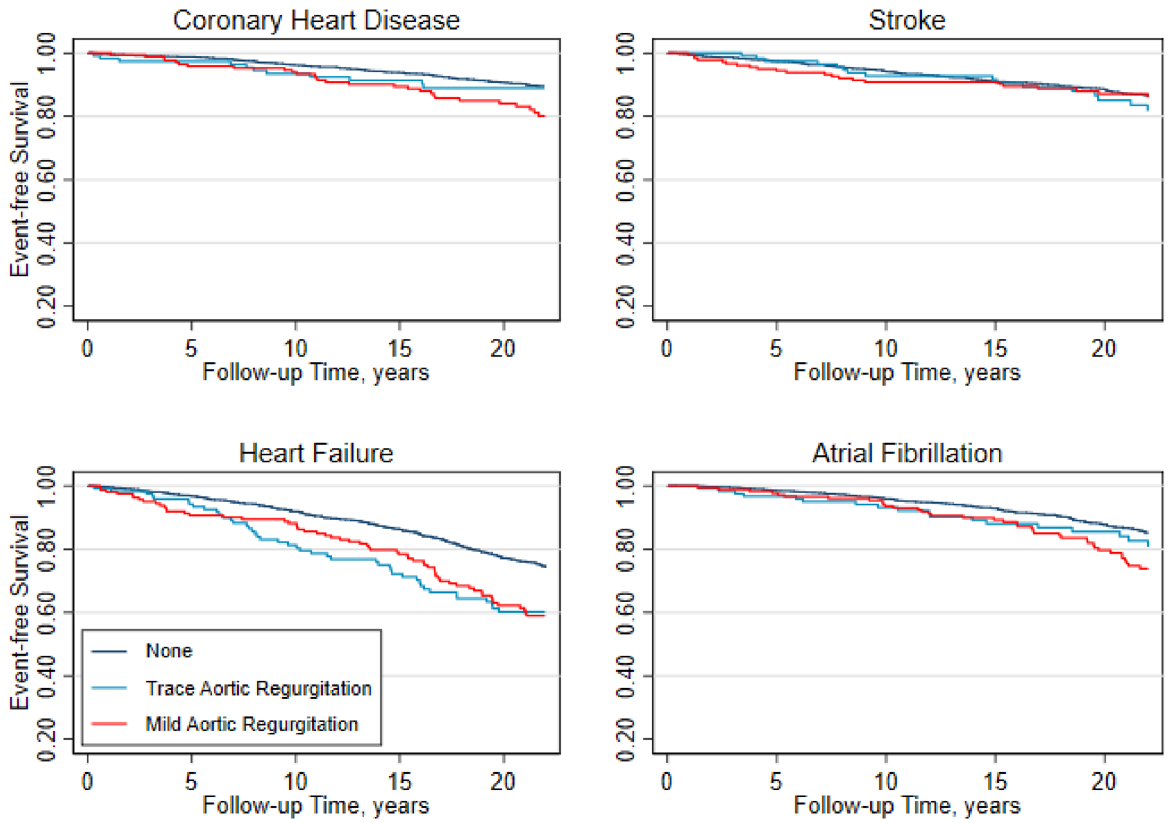

© 2022 Matsushita K et al. *JAMA Network Open.*

eFigure 4. Kaplan-Meier Survival Estimates of Cardiovascular Outcomes by Mitral Regurgitation

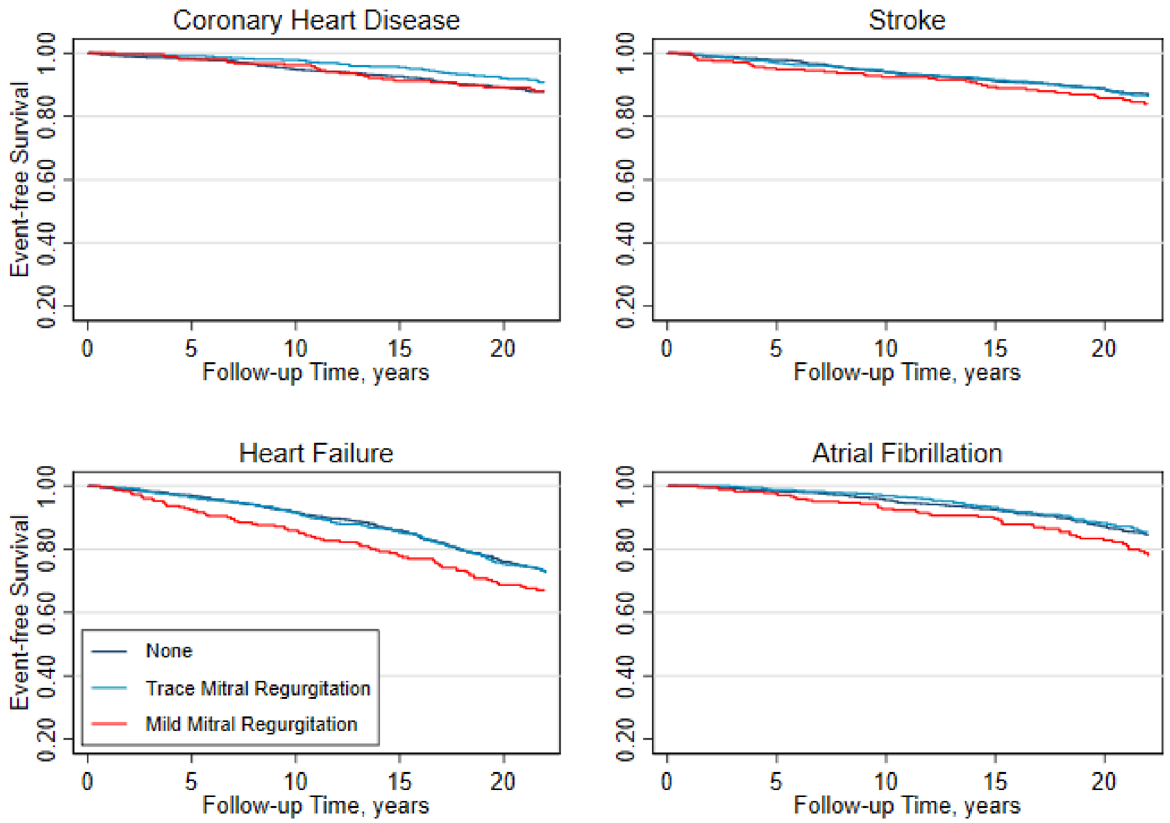

© 2022 Matsushita K et al. *JAMA Network Open.*

eFigure 5. Kaplan-Meier Survival Estimates of Cardiovascular Outcomes by the Number of Valvular Lesions

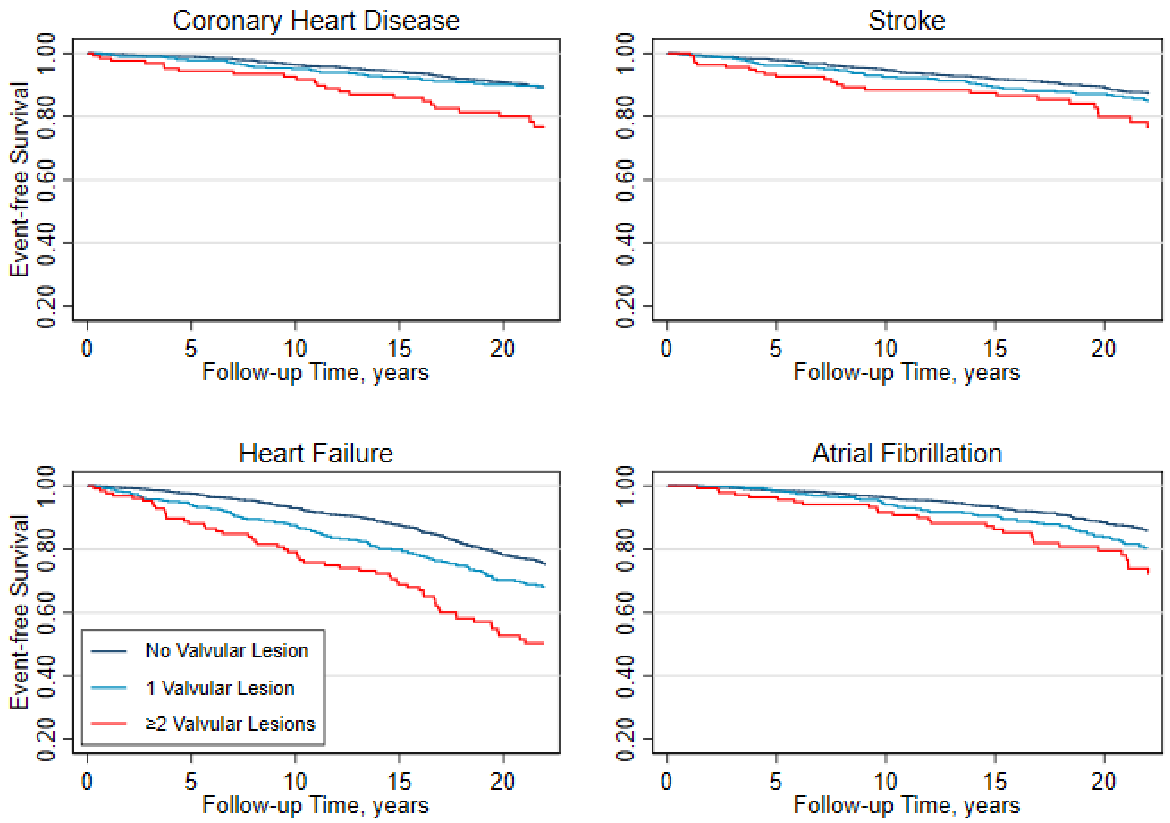

© 2022 Matsushita K et al. *JAMA Network Open.*
